# Brucellosis mimicking upper gastrointestinal symptoms: A diagnostic challenge—A rare case report

**DOI:** 10.1016/j.idcr.2026.e02715

**Published:** 2026-07-29

**Authors:** Zahra Montaseri, Maryam Montaseri, Mohammad Ekrahi, Mohammadreza Eskandari Kooshkeghazi

**Affiliations:** a Department of Infectious Diseases, School of Medicine, Fasa University of Medical Sciences, Fasa, Iran; b Department of Food Hygiene and Public Health, School of Veterinary Medicine, Shiraz University, Shiraz, Iran; c Student Research Committee, Fasa University of Medical Sciences, Fasa, Iran

**Keywords:** Brucellosis, Gastritis, Nonspecific presentation, Case report

## Abstract

Although brucellosis is a common zoonotic disease with multisystem involvement, presentations dominated by chronic upper gastrointestinal symptoms are exceptionally rare and may pose a diagnostic challenge. A 20-year-old woman from Fars Province, Iran, presented with a 6-month history of anorexia, nausea, vomiting, epigastric pain, mild myalgia, and an unintentional 10-kg weight loss, without fever, arthralgia, or night sweats. Upper gastrointestinal endoscopy revealed Los Angeles Grade A esophagitis and erosive antral gastritis, while gastric biopsy was negative for *Helicobacter pylori.* Routine laboratory investigations, biochemical tests, and imaging studies were unremarkable except for thalassemia minor. A detailed exposure history revealed consumption of unpasteurized sheep colostrum. Serological testing and blood PCR targeting the BCSP31 gene confirmed brucellosis. The patient responded well to standard anti-brucellosis therapy, with complete resolution of symptoms during the 12-month follow-up*.* To the best of our knowledge, this is the first reported case of microbiologically confirmed brucellosis presenting predominantly with chronic upper gastrointestinal symptoms accompanied by endoscopic erosive gastritis. This exceptionally uncommon presentation highlights the importance of considering brucellosis in the differential diagnosis of persistent and unexplained upper gastrointestinal symptoms in endemic regions, particularly in patients with relevant epidemiological exposures, to avoid delayed diagnosis and inappropriate management.

## Introduction

Brucellosis is a significant global zoonotic disease, particularly in endemic regions such as the Middle East, including Iran. Humans may be infected through direct contact with infected livestock or consumption of unpasteurized dairy products. Brucellosis can affect various organ systems such as musculoskeletal, hematological, digestive, nervous and urinogenital systems [1]. Brucellosis can present with a wide range of nonspecific manifestations. The most frequent symptoms include fever, arthralgia, myalgia, fatigue, and sweating. However, gastrointestinal symptoms are diverse and may occasionally predominate in the clinical presentation of brucellosis. These symptoms range from relatively mild (e.g., anorexia, diarrhea, or constipation) to serious complications (e.g., mesenteric lymphadenitis, liver or spleen involvement, cholecystitis, colitis, pancreatitis, and peritonitis) [1], [2], [3], [4]. Gastrointestinal manifestations occurring without prominent classical systemic features have been reported relatively infrequently; nevertheless, they vary in severity and may be overlooked [1], [2], [5]. Predominant upper gastrointestinal presentations associated with endoscopic gastritis are particularly uncommon.

This study presents a case of microbiologically confirmed brucellosis in a patient whose clinical presentation was dominated by chronic upper gastrointestinal symptoms, including anorexia, nausea, vomiting, epigastric pain, and substantial weight loss, together with mild myalgia and endoscopic erosive antral gastritis.

## Case presentation

A 20-year-old woman from Fars Province, Iran, presented to our hospital on June 24, 2025, with a 6-month history of anorexia, nausea, vomiting, epigastric pain, mild myalgia, and an unintentional 10-kg weight loss. Her symptoms had progressively worsened without periods of remission. She had no history of fever, arthralgia, or night sweats. She was a known case of thalassemia minor and had been taking oral folic acid (1 mg/day).

On admission, her vital signs were stable, and physical examination was unremarkable except for epigastric tenderness. During the preceding six months, she had been hospitalized several times at different medical centers because of persistent gastrointestinal symptoms. Laboratory investigations, including complete blood count, serum biochemistry, coagulation profile, inflammatory markers, hormonal assays, and routine serological tests, were within normal limits except for findings consistent with thalassemia minor (Table 1). Cardiac evaluation, including electrocardiography and qualitative troponin testing, was unremarkable.

**Table 1:** The results of biochemical, hematological, Serological and immunological, coagulation, hormone, inflammatory tests performed for the patient during last 6 months of the disease.

**Biochemical test**			**Haematological test**		
Factor	Result (unit)	Normal range	Factor	Result (unit)	Normal range
Blood Sugar	91 (mg/dL)	70 – 99	Hb	10.9 (g/dl)	12 – 16
BUN	8 (mg/dL)	6 – 12	HCT	32.8 (%)	38 – 47
Creatinine	0.9 (mg/dL)	0.6 – 1.3	MCV	56.6 (fL)	80 – 96
Sodium (Na)	135 (mEq/L)	135 – 145	MCH	18.9 (pg)	27 – 32
Potassium (K)	3.6 (mEq/L)	3.5 – 5.2	MCHC	33.2 (g/dl)	32 – 36
Total Bilirubin	0.57 (mg/dL)	0.1 – 1.2	RDW.C	16.8 (%)	11.5 – 14.5
Direct Bilirubin	0.20 (mg/dL)	0 – 0.3	RDW.S	34 (fL)	40 – 55
AST (SGOT)	14 (U/L)	Up to 37	Platelets	182 (10 ³ /µL)	150 – 450
ALT (SGPT)	9 (U/L)	Up to 40	MPV	10.6 (fL)	8.6 – 12.6
Alkaline Phosphatase	141 (U/L)	64 – 306	**Serology and immunology test**		
Total Protein	7.2 (g/dL)	6.4 – 8.3			
Albumin	4.1 (g/dL)	3.5 – 5.2	Tissue Transglutaminase Ab	6.1 (U/ml)	Normal < 12
Globulin	3.1 (g/dL)	2.3 – 3.5	Anti-Endomysial Ab (IgA)	15.8 (U/ml)	Negative < 20
Serum Iron	48 (mcg/dL)	37 – 165	ANA Detect (Alegria)	0.3 (Index)	Negative < 1
TIBC	295 (mcg/dL)	250 – 450	Anti-dsDNA Screen (Alegria)	8.4 (U/ml)	Negative < 25
Iron Saturation	30 (%)	20 – 50	Anti B2GP IgG	0.06 (U/ml)	Negative < 5
Serum Ferritin	110 (ng/ml**)**	20 – 120	Anti B2GP IgM	1.10 (U/ml)	Negative < 5
Folic Acid	14.8 (ng/ml)	4.5 – 17	ACLA Screen (Alegria)	2.5 (U/ml)	Negative < 10
Vitamin B12	283 (pg/ml)	190 – 860	ACLA IgM (Alegria)	0.70 (U/ml)	Negative < 7
Amylase	48 (IU/L)	0 – 100	Wright	1/160	>1/80
Lipase	35 (IU/L)	0 – 60	2-mercaptoethanol (2ME)	1/80	> 1/40
Calcium	9.8 (mg/dL)	8.3 – 10.5	**Coagulation test**		
Phosphorus	3.4 (mg/dL)	3.2 – 5.6	Lupus anticoagulant (DRVV-Screen)	37.8 (Sec)	28 – 43
Magnesium	2.1 (mg/dL)	1.9 – 2.5	**Hormone test**		
Zinc	104 (mcg/dL)	70 – 114	Cortisol AM	9.5 (µg/dl)	5 – 23
Vitamin D3 (25-OH)	30.2 (ng/ml)	>20	TSH	1.53 (µIU/ml)	0.51 – 4.3
**Haematological test**			**Inflammatory test**		
WBC	9.2 (10 ³ /µL)	4 – 11	CRP	3 (mg/L)	< 6
RBC	5.8 (10⁶ /µL)	4.2 – 5.4	ESR	8 (mm/h)	0–29

Evaluation for *Helicobacter pylori* infection, including serum IgM antibody (0.66 U/ml), stool antigen, and urea breath tests, was negative. Upper gastrointestinal endoscopy revealed Los Angeles Grade A esophagitis, a Grade I sliding hiatal hernia, and erosive antral gastritis. Gastric biopsy showed no evidence of glandular atrophy, intestinal metaplasia, loss of parietal cells, or *H. pylori* infection. The biopsy was not specifically evaluated for *Brucella* by culture, PCR, or other organism-specific methods. So, direct gastric mucosal infection was not confirmed for brucellosis. Abdominal and pelvic ultrasonography, contrast-enhanced computed tomography of the abdomen and pelvis, and brain magnetic resonance imaging showed no abnormalities. The patient denied the use of nonsteroidal anti-inflammatory drugs, corticosteroids, or alcohol.

**Fig. 1:**
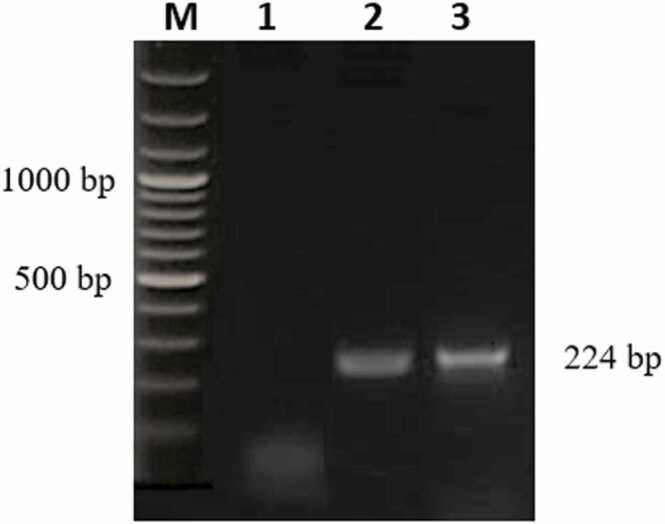
The gel electrophoresis of PCR products (224 bp) for the *Brucella* spp. *BCSP31* gene. M: 100 bp DNA ladder, Lane 1: Negative control, Lane 2: Positive sample, Lane 3: Positive control.

Further history-taking revealed regular consumption of unpasteurized sheep colostrum during the months preceding symptom onset. Given this epidemiological exposure, brucellosis was suspected. Serological testing demonstrated a Wright agglutination titre of 1:160 and a 2-mercaptoethanol (2ME) titre of 1:80, both considered positive according to the Iranian National Brucellosis Guidelines [6], [7]. To confirm the diagnosis, PCR targeting the *BCSP31* gene was performed on the serologically positive blood sample. Genomic DNA was extracted using a commercial kit (Cinnagen, Iran), and amplification was carried out using B4 (5′-TGGCTCGGTTGCCAATATCAA−3′) and B5 (5′-CGCGCTTGCCTTTCAGGTCTG−3′) primers with an annealing temperature of 60°C [8]. PCR yielded the expected 224-bp amplicon, confirming *Brucella* infection (Fig. 1).

The patient was treated with doxycycline (100 mg twice daily) and rifampin (300 mg twice daily) for 8 weeks, together with intramuscular streptomycin (1 g/day) for the first 3 weeks. A proton-pump inhibitor was also administered for 8 weeks. Notably, the patient had previously received proton-pump inhibitor therapy for her persistent upper gastrointestinal symptoms before the current admission, without meaningful or sustained improvement. No structured dietary intervention or other specific long-term treatment for gastritis was prescribed. Her gastrointestinal symptoms improved markedly, and she was discharged after 10 days of hospitalization. At the 12-month follow-up, she remained asymptomatic, with complete resolution of gastrointestinal complaints and no evidence of disease relapse.

**Fig. 2:**
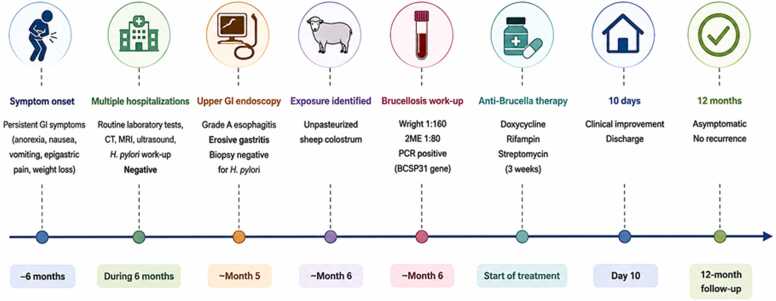
Clinical timeline illustrating the patient's symptom progression, diagnostic investigations, confirmation of brucellosis, antimicrobial treatment, and clinical outcome over a 12-month follow-up.

For clarity, the patient's clinical course from symptom onset to diagnosis, treatment, and 12-month follow-up is summarized in the timeline shown in Fig. 2

## Discussion

This study describes a case of brucellosis presenting predominantly with chronic upper gastrointestinal manifestations, substantial unintentional weight loss, mild myalgia and endoscopic erosive antral gastritis. The condition remained undiagnosed for six months because of its nonspecific presentation. Brucellosis has a wide spectrum of clinical presentations. The most common manifestations are fever, fatigue, hyperhidrosis, and large-joint arthralgia [1], [5]. However, depending on the organs involved and the stage of the disease, brucellosis may present with atypical symptoms, including gastrointestinal manifestations. Gastrointestinal manifestations of brucellosis range from mild presentations (e.g., anorexia, nausea, vomiting, abdominal pain, and diarrhea) to severe complications (e.g., mesenteric lymphadenitis, hepatitis, and splenomegaly) [4]. Although gastrointestinal manifestations are relatively common, they usually occur in combination with systemic symptoms rather than as the predominant clinical presentation.

In the present case the patient presented a broader clinical syndrome dominated by chronic upper gastrointestinal symptoms, including anorexia, nausea, vomiting, epigastric pain with mild musculoskeletal involvement and marked weight loss; however, no fever, arthralgia, night sweats, or other classical manifestations of brucellosis were observed. Anorexia, reported in 22–45% of patients, is a common presentation in brucellosis [4]. The assumed pathophysiology may involve the effects of inflammatory cytokines, such as IFN-γ and TNF-α, on the appetite center within the ventromedial hypothalamus [9]. Vomiting, a nonspecific complaint, may result from cytokine-mediated inflammation associated with brucellosis [4].

There are few case reports of brucellosis presenting predominantly as gastroenteritis [5], [10]. For example, Salih and Alothman reported a 21-year-old man presenting with fever, vomiting, diarrhea, leukopenia, thrombocytopenia, and elevated liver enzymes. Another case involved a 68-year-old woman with night sweats, anorexia, colicky abdominal pain, diarrhea, ileitis, and mild splenomegaly [11]. Wang et al. (2024) reported a 69-year-old man with intestinal perforation, acute diffuse peritonitis, myalgia, and arthralgia [5]. Unlike these previously reported cases, which showed intestinal involvement with or without hepatosplenic manifestations, our patient presented predominantly with chronic upper gastrointestinal symptoms and endoscopic erosive antral gastritis in the absence of fever, hepatosplenomegaly, or other systemic features. To the best of our knowledge, this represents the first microbiologically confirmed case of brucellosis presenting predominantly with chronic upper gastrointestinal symptoms. The mechanism underlying the association between brucellosis and the gastric findings in this patient remains unclear. Proposed mechanisms include the ability of *Brucella* spp. to survive gastric acidity, invade epithelial cells and macrophages, and induce local and systemic inflammatory responses through cytokines such as TNF-α, IL-1β, IL-6, and IFN-γ, ultimately leading to gastric inflammation [12], [13], [14]. The cytokine-mediated inflammatory response may therefore contribute to the development of gastritis in affected patients. Nevertheless, because microbiological confirmation was obtained from blood rather than gastric tissue, definitive direct gastric involvement was not assessed in this study. Therefore, the findings support a temporal and clinical association between brucellosis and the predominantly gastrointestinal presentation.

Since persistent upper gastrointestinal symptoms and endoscopic gastritis are nonspecific findings shared by many gastrointestinal disorders, it often leads to misdiagnosis. In this case, despite multiple hospitalizations and extensive diagnostic evaluations, brucellosis remained undiagnosed for six months. This pattern of extensive investigation for nonspecific symptoms is characteristic of brucellosis, leading to delayed diagnosis ranging from weeks to months [15], [16]. Although brucellosis is endemic in Iran, diagnostic delays continue to occur [17]. The diagnostic delays may be due to the fact that brucellosis is known as the great mimicker, capable of presenting with diverse clinical manifestations that resemble many infectious and noninfectious diseases [18]. Atypical or nonspecific symptoms together with challenges in laboratory diagnosis may further contribute to delayed diagnosis, especially in endemic regions. Delayed diagnosis and treatment increase the risk of disease complications and therapeutic failure [17], [18].

Besides *H. pylori* infection, the differential diagnosis of chronic or erosive gastritis includes autoimmune gastritis, medication- or chemical-induced reactive gastropathy, bile reflux, alcohol-related mucosal injury, and less common eosinophilic, lymphocytic, granulomatous, and infectious forms of gastritis [19]. In the present case, the patient denied the use of nonsteroidal anti-inflammatory drugs, corticosteroids, and alcohol. Histopathological examination showed no glandular atrophy, intestinal metaplasia, or loss of parietal cells, making advanced autoimmune atrophic gastritis less likely. However, a limitation of this case is that uncommon immune-mediated, infectious, and reactive causes were not comprehensively evaluated using dedicated diagnostic tests. Therefore, although several common causes were not identified, a concomitant alternative cause of the gastric findings cannot be completely excluded.

Given that ingestion of unpasteurized dairy products or contact with infected animals are the main routes of transmission [20], obtaining a detailed exposure history is essential when evaluating patients with unexplained gastrointestinal symptoms in endemic areas. In the present case, the history of consuming unpasteurized sheep colostrum prompted targeted serological and molecular testing, ultimately leading to the correct diagnosis of systemic brucellosis.

## Conclusion

Although gastrointestinal involvement is recognized in brucellosis, a clinical presentation dominated by chronic upper gastrointestinal symptoms and accompanied by endoscopic erosive gastritis is exceptionally uncommon. Although direct microbiological gastric involvement was not assessed, the positive blood PCR and serological findings, together with the predominant gastrointestinal presentation, endoscopic erosive gastritis, and complete clinical response to anti-brucellosis therapy, support a probable association between brucellosis and the gastrointestinal manifestations.

This case highlights how atypical and predominantly gastrointestinal presentations can lead to prolonged diagnostic delays, repeated hospitalizations, and unnecessary investigations. In endemic regions, clinicians should consider brucellosis in the differential diagnosis of persistent, unexplained upper gastrointestinal symptoms, particularly in patients with relevant epidemiological exposures such as consumption of unpasteurized dairy products. Early recognition, supported by appropriate serological and molecular testing, may facilitate timely diagnosis and appropriate management.

## CRediT authorship contribution statement

**Zahra Montaseri:** Writing – review & editing, Visualization, Validation, Methodology, Conceptualization. **Mohammad Ekrahi:** Writing – review & editing, Validation, Methodology. **Maryam Montaseri:** Writing – review & editing, Writing – original draft, Visualization, Validation, Methodology. **Mohammadreza Eskandari Kooshkeghazi:** Writing – review & editing, Investigation, Data curation.

## Ethics declaration

Written informed consent to take part in the study and to publish the article has been obtained from all participants or their legal representatives. The privacy rights of participants have been observed.

This study was performed in compliance with relevant laws, regulatory frameworks and guidelines where the research took place. This study was approved by the Fasa University Medical Science (Approval No. IR.FUMS.REC.1404.162).

This research follows the CARE guidelines and the CARE checklist.

## Ethical approval

All procedures performed in studies involving human participants were in accordance with the ethical standards Fasa University Medical Science (IR.FUMS.REC.1404.162) and with the 1964 Helsinki declaration and its later amendments or comparable ethical standards.

## Informed consent

Written informed consent for publication of this case report was obtained from the patient and hospital record.

## Funding

The authors received no financial support for the research, authorship, or publication of this article.

## Declaration of Competing Interest

The authors declare no conﬂicts of interest in this study.
